# P-1313. Large Outbreak of *Staphylococcus aureus* and *Bacillus cereus* Caused by Ready-to-Eat Meals in Aomori, Japan

**DOI:** 10.1093/ofid/ofae631.1494

**Published:** 2025-01-29

**Authors:** Mizue Eita, Yuichiro Yahata, Natsuko Nakamura, Keiichiro Fujita, Masato Saito, Kaori Matsukura, Hiromi Takagi, Mizuki Nakamura, Tomoe Shimada, Atsuko Ishii, Masanobu Kudou, Tomimasa Sunagawa

**Affiliations:** National Institute of Infectious Diseases, chiyoda-ku, Tokyo, Japan; National Institute of Infectious Diseases, chiyoda-ku, Tokyo, Japan; National Institute of Infectious Deaseases, chiyoda-ku, Tokyo, Japan; Hachinohe City Public Health Center, Hachinohe-shi, Aomori, Japan; Hachinohe City Public Health Center, Hachinohe-shi, Aomori, Japan; Hachinohe City Public Health Center, Hachinohe-shi, Aomori, Japan; Hachinohe City Public Health Center, Hachinohe-shi, Aomori, Japan; Hachinohe City Public Health Center, Hachinohe-shi, Aomori, Japan; National Institute of Infectious Deaseases, chiyoda-ku, Tokyo, Japan; Hachinohe City Public Health Center, Hachinohe-shi, Aomori, Japan; Hachinohe City Public Health Center, Hachinohe-shi, Aomori, Japan; National Institute of Infectious Diseases, chiyoda-ku, Tokyo, Japan

## Abstract

**Background:**

Ready-to-eat meals are a vehicle of multijurisdictional outbreaks of foodborne diseases worldwide. *Ekiben* is a type of ready-to-eat meal commonly sold at train stations and occasionally at markets in Japan. On September 17, 2023, the Hachinohe City Public Health Center (HCPHC) received numerous complaints from consumers in several prefectures who had eaten *ekiben* produced by Company A in Hachinohe, Aomori, Japan.

**Methods:**

In collaboration with HCPHC, we conducted an epidemiological investigation, traceback/traceforward investigation, and an inspection of Company A. The local public health authorities performed microbiological tests of food and stool samples.

**Results:**

A total of 554 symptomatic cases were identified in 29 prefectures. The highest proportion of symptoms was diarrhea (86%), followed by vomiting (61%), nausea (59%), and stomachache (51%). Company A produced 67 lunchboxes of which 23 types of lunchboxes occurred cases. 23 lunchboxes contained seafood as an ingredient but no common ingredients except for rice. The rice was self-produced and/or outsourced from Company B. Company A received triple the number of usual orders from retailers and trading companies due to an “Ekiben Fair” held on September 15 and 16. Company A could not prepare enough steamed rice for such an event on its own, so it outsourced the production of some rice to Company B, which shipped the rice at temperatures ranging from 42°C–49°C. *Staphylococcus aureus* with enterotoxin and *Bacillus cereus* with enterotoxin were detected from stool samples as well as steamed rice and other food samples with sufficient amounts of bacteria to cause the disease. Company A did not have an appropriate HACCP plan, operation manual, response plan, or processing records. The company had no records of hygiene training, hygiene education, health checks, or hand-injury records for its temporary workers. We concluded that the likely causative food was steamed rice that had been shipped at an improper storage temperature, and processing the lunchboxes.

**Conclusion:**

We recommended that Company A store rice at the proper temperature, implement an appropriate HACCP plan, create a standard operation manual, maintain processing records, separate clean and contaminated areas, and prepare a response plan in case of unusual situations.

**Disclosures:**

**All Authors**: No reported disclosures

